# Migration Influences on the Allostatic Load of Children: Systematic Review Protocol

**DOI:** 10.2196/resprot.8332

**Published:** 2018-01-30

**Authors:** Ivan Neil Gomez, Cynthia YY Lai, Trevor WK Yung, Chetwyn CH Chan, Hector WH Tsang

**Affiliations:** ^1^ Department of Rehabilitation Sciences Faculty of Health and Social Sciences The Hong Kong Polytechnic University Kowloon China (Hong Kong); ^2^ Center for Health Research and Movement Science College of Rehabilitation Sciences Univesity of Santo Tomas Manila Philippines

**Keywords:** migration, allostatic load, allostasis, children

## Abstract

**Background:**

Migration is a worldwide phenomenon in recent times. Recently, documented studies suggest that the change in environments involved in migration may have an influence on children’s allostatic load related to health and well-being.

**Objective:**

The aim of this review is to systematically search the extant literature and critically examine the evidence on how migration affects allostatic load in children and describe the relevant methods in measuring allostatic load.

**Methods:**

A systematic review will be conducted to recapitulate the evidence on the influence of migration on allostatic load and describe the methods employed in measuring allostatic load parameters among migrant children using the following search terms combinations: 1) allostasis OR allostatic OR allostatic load OR allosta*; 2) migration OR migrant OR immigration OR immigrant OR migra* OR *migra*; and 3) children OR child* OR adolescen*. We will search for peer-reviewed articles in English using a three-step process: title and abstract review, individual article review, and reference hand-searching among the following databases: Medline, CINAHL, ProQuest, PubMed, Science Direct and BioMed Central. Two independent review authors will analyze for data quality, level of evidence and risk of bias; a third review author will be consulted if consensus cannot be met. Data on study details, participant characteristics, allostatic load operationalization and description, methods, and results summary will be extracted. Evidence will be synthesized statistically when possible and narratively clustered into themes.

**Results:**

At present, we have conducted only a preliminary search to test out our search terms. The systematic search, appraisal, synthesis and analysis will be finished by June 2018. It is projected that the manuscript that describes the systematic review will be available by the last quarter of 2018.

**Conclusions:**

The results of this systematic review have implications on supporting the concept of allostasis as a mechanism underlying the adaptive processes related to migration. Furthermore, our findings can lead to the development of innovative evidence-informed evaluation and intervention programs aimed at migrant children's needs. Likewise, it is hoped that this review can be an impetus to inform health and sociopolitical policies responsive of migrant children's current contexts.

**Trial Registration:**

International Prospective Register of Systematic Reviews (PROSPERO): CRD42017068895; https://www.crd.york.ac.uk/prospero/display_record.php?RecordID=68895 (Archived by WebCite at http://www.webcitation.org/6wprRkxvA)

## Introduction

Different areas of inquiry have tackled migration from their own points of view. Herein, we look at migration from a biological perspective, where migration is generally defined as the movement of an individual or group of individuals from one territory to another to establish a new area of residence [1,2]. This entails a change in the environment with respect to one's place of origin and host. The concept of allostasis is intended to be used in this review as a unifying concept to explain the underlying mechanism related to the adaptive processes of migration and its effect on the health and well-being of children. The concept of allostasis stemmed from the earlier research of McEwen [3,4], Sterling and Eyer [5], and McEwen and Wingfield [6]. In allostasis, alteration of the regulatory parameters allows a person to adapt to environmental challenges through the development of a new baseline to maximize the individual’s performance [7,8]. Allostasis is responsible for short-term adaptation, survival, and homeostasis, but according to McEwen and Wingfield [6], it can potentially produce permanent changes, termed allostatic load, in a person in prolonged exposures. Allostasis has been widely used to explain the adaptation of migrating animals (ie, birds, fishes) to their environments of habitation using physiological parameters related to the autonomic or neuroendocrine mechanisms [9,10]. Results are congruent in the fact that physiological parameters are regulated upon immigration, and this is related to the survival in the site of resettlement. Among human studies, allostatic load parameters were used to represent regulation of various physiological systems (ie, cardiovascular, metabolic, body composition) using several biomarkers among immigrant adults [11,12,13]. However, as of date, the evidence on the influence of migration on the allostatic load in children has yet to be systematically reviewed.

The allostasis model suggests the interaction mechanisms between individual differences, behavioral responses and physiological responses contextualized within a particular environment can reflect the vulnerability or adaptive functions of individuals [14]. Taken together, the interacting roles of each can presume a child’s overall health and well-being. Individual differences may refer to demographic characteristics of individuals (ie, gender, age, ethnicity, genetic predispositions, environment, sociocultural influences, socioeconomic status, family dynamic, place of birth, site of resettlement), which has long been proven to affect the allostatic load [4]. The role of the environment is greatly emphasized in the allostasis model, having the capacity to influence both behavioral and physiological response. The physical environment (ie, temperature, humidity, seasonality, noise, physical landscapes and features) needs to be succinctly accounted for when employing physiological outcomes [15,16,17,18]. Emerging evidence suggests that nativity or place of birth influences the allostatic load [19]. Previous research has likewise implicated sensory-related, temperament and resilience behaviors as possible factors that can shape allostasis [20,21,22,23,24]. The classic paper of Berry [25] provided foundational insight on the factors that can support adaptation among immigrants and has mentioned similar factors. However, these factors mainly focused on psychological adaptation to a new culture and cannot account for salient biological changes related to migration.

In 2015, there were 244 million recorded international migrants, with 20% under the age of 20 [26]. The impact of migration on health has well been studied previously, and recent evidence suggests related long-term physical and mental health issues [27]. Specifically, children ages 6 to 17 may be at risk for psychosocial problems that can have deleterious effects on their health and well-being [28]. However, most of the research done focused mainly on the effects of immigration rather on latent mechanisms which may explain such effects. The concept of allostasis may offer a novel perspective on how a change in the environment influences the regulation of behavior and physiological responses among children, and a better understanding of the mechanisms underlying adaptation, or maladaptation, of migrant children in order to provide relevant healthcare programs and interventions.

In light of the lack of existing systematic reviews, contemporary gaps in knowledge, and the novel application of the concept of allostasis on human migration, the authors of this review seek to systematically review the extant literature that examines how migration influences the allostatic load among children. Specifically, this review aims to answer the following research questions:

1. What is the evidence on the influence of migration on allostatic load among children?
2. What are the allostatic load measures relevant to migrant children?
3. What methods are usually employed in measuring allostatic load parameters among children?

## Methods

The proposed systematic review aims to recapitulate the evidence on the influence of migration on allostatic load; summarize the commonly used allostatic load measures; and describe the methods employed in measuring allostatic load parameters among migrant children. This review is registered on the PROSPERO database with registration number: CRD42017068895, developed and reported based on the Preferred Reporting Items for Systematic Review and Meta-Analysis Protocols (PRISMA-P) guidance [29]. The self-assessed PRISMA-P checklist for this protocol is shown in Multimedia Appendix 1.

### Study Selection

This systematic review will consider studies for inclusion based on the following criteria.

#### Types of Studies

This review will consider designs including before and after studies, prospective and retrospective cohort studies, case-control studies and analytical cross-sectional studies for inclusion. Furthermore, we will also consider descriptive epidemiological study designs including case series, individual case reports and descriptive cross-sectional studies for inclusion. In the absence of research studies, other text such as opinion papers and reports will be considered. The reviewed studies will be summarized based on their typology.

#### Types of Participants and Exposures

This study will primarily consider studies include migrant subjects between the ages of 2 to 18 years old from typically-developing populations regardless of gender, ethnicity/race, and country of origin/migration, with no known medical, neurobehavioral or psychological history. In this paper, a child will be considered an individual under the age of 18 years old [30]. The lower bound limit was set at age 2 years old to differentiate these groups specifically from infants [31]. In cases, where a clinical group is presented with or without a comparison normative group, the data from these studies will likewise be reported in the final documentation.

The exposure of interest shall be “migration.” Several definitions suggest that migration can be classified as either internal or external migration; while migrants can be further categorized as native-born or foreign-born. Due to the exploratory nature of this review, we will consider all of these types of migration exposures and consequently report these data, as well as the operational definitions used in the reviewed studies. However, excluded in the migration category are those results that have experienced forced migration (ie, refugees, victims of famine, disasters, etc). Previous research suggests that adverse life events in childhood may have an influence on a child’s neurobehavioral, psychophysiological or physiological development [32,33,34]. Thus, only types of voluntary migration are considered.

#### Types of Outcomes

Part of the aim of this study is to summarize the common outcome measures to conceptualize allostatic load parameters. Based on the concept of allostasis, we will classify the outcome measures into two categories: 1) behavioral measures (ie, temperament, personality, resilience, health-seeking behaviors, etc); and 2) physiological measures (ie, heart rate, body mass index [BMI], height, weight, etc). Physiological measures may also be in the form of neurobiological or neurophysiological indices (ie, cortisol, hormones, heart rate variability, etc). Data from other outcomes not included in this protocol but emerges from this review shall be succinctly discussed in the final report.

### Literature Search

#### Search Strategy

Our search strategy aims to find peer-reviewed published studies. A three-step search strategy will be utilized in this review. An initial limited search of the included databases will be undertaken followed by analysis of the text words contained in the title and abstract, and of the index terms used to describe an article by the primary author. A second search using all identified keywords and index terms will then be undertaken across all included databases. Lastly, a reference-searching strategy will be entailed among the primary studies that have gone through the second search level.

For this review, we expanded the search strategy to capture all possible studies using the following databases: Medline, CINAHL, ProQuest, PubMed, Science Direct and BioMed Central. This will be followed by an analysis of the text words contained in the title and abstract and of the index terms used to describe the article. The following databases were chosen based on the recommendation of an experienced librarian in the field of health sciences who likewise helped in validating the search terms of this review paper. The chosen databases include other databases within its system of resources, thus increasing the platform where the search strategy is employed. Studies published in English or which have an English version will be considered for inclusion in this review. The search data will range from January 2007 to December 2017. Initial keywords to be used will be: allostasis OR allostatic OR allostatic load OR allosta*; 2) migration OR migrant OR immigration OR immigrant OR migra* OR *migra*; and 3) children OR child* OR adolescen*. The use of “*” represents truncated terms implemented to increase the scope of the search for articles. This review will likewise consider dissertation papers that meet the previously mentioned inclusion criteria, provided these are appropriately indexed within the abovementioned databases and retrieved during the search. An example of search strategy and results conducted in MEDLINE is shown in Multimedia Appendix 2.

Furthermore, this review will entail contacting authors of the initially identified articles, reference list hand-searching and considering grey literature (ie, existing data sets, unpublished data, theses and dissertations, technical reports) to widen the scope of the search.

#### Screening and Review Process

Full-text articles retrieved from the three-step search strategy and hand-searched additional articles will be screened and reviewed by two independent review authors for quality appraisal, levels of evidence and risk of bias for potential inclusion in this review. In the event of a disagreement between the two independent review authors, a meeting will be held to reach a consensus; but if a consensus cannot be reached, an independent third reviewer will be consulted. An example of the sample flow diagram of study selection procedures is shown in Multimedia Appendix 3.

#### Data Quality and Level of Evidence

Papers selected for retrieval will be assessed by two independent reviewers for methodological validity prior to inclusion in the review using standardized critical appraisal instruments from the Joanna Briggs Institute Meta-Analysis of Statistics Assessment and Review Instrument (JBI- MAStARI) [35]. The JBI-MAStARI is a comprehensive set of tools that guides researchers in the conduct and preparation of high-quality systematic reviews [35]. The JBI MAStARI appraisal tools are shown in Multimedia Appendix 4.We will use the NHMRC Evidence Hierarchy [36] to assign designations of “levels of evidence” according to the type of research question of the studies appraised. The NHMRC Evidence Hierarchy is shown in Multimedia Appendix 5. Any disagreements that arise between the two assigned independent reviewers will be resolved through discussion or with a third reviewer.

#### Data Extraction

Quantitative data will be extracted from papers included in the review using the relevant standardized data extraction tool from the Joanna Briggs Institute Meta-Analysis of Statistics Assessment and Review Instrument [35]. The data extraction tool to be used in this research is shown in Multimedia Appendix 6. The same review authors that screened and reviewed the primary articles for review shall extract the following data:

1. Study Details: type of study and whenever appropriate, the method of randomization and presence or absence of blinding;
2. Demographic Characteristics: age, gender, ethnicity/race, socio-economic status, number of participants recruited, number of drop-puts/withdrawals whenever possible;
3. Definition and operationalization of allostatic load;
4. Type of outcome measures: behavioral or physiological measures;
5. Details of outcome measure procedures: instrumentation, procedures, methods;
6. Results: details on the role of migration on the autonomic state as it influences child behavior.

When disagreements arise related to data extraction, a discussion will be conducted to reach a consensus on a decision. However, if a consensus cannot be reached, a third review author will be consulted. In the case of missing, incomplete or incomprehensive data, the authors of the reviewed papers will be contacted by email for clarifications and supplement of needed information whenever possible.

#### Risk of Bias

For assessing the risk of bias within studies, this review will adopt the Cochrane “Risk of bias” [37] tool which included the following criteria:

1. Sequence generation;
2. Allocation concealment;
3. Blinding of participants, personnel and outcome measures;
4. Incomplete outcome data, and;
5. Selective outcome reporting.

The risk of bias tool to be used in this research is shown in Multimedia Appendix 7. Furthermore, the risk of bias across studies shall be reported using the abovementioned criteria from the Cochrane [37] group, with additional criteria added in response to other authors’ recommendations [38,39]. On this basis we added two criteria, “Outcome Measure Processes” and “Age Inclusions” using the thresholds for across studies judgment as suggested [39]:

1. Low risk of bias—“Most information is from studies at low risk of bias.”
2. Unclear risk of bias—“Most information is from studies at low or unclear risk of bias.”
3. High risk of bias—“The proportion of information from studies at high risk of bias is sufficient to affect the interpretation of the results.”

Assessment of risk of bias shall be undertaken by two review authors where discrepancies are identified and resolved through discussion to reach a consensus on results, while a third author will be consulted in case a consensus cannot be met.

### Analysis

#### Statistical Analysis

Quantitative papers, when possible, will be pooled in a statistical meta-analysis using JBI-MAStARI [35]. All results will be subject to double data entry. Effect sizes expressed as odds ratio (for categorical data) and weighted mean differences (for continuous data) and their 95% CI will be calculated for analysis. Heterogeneity will be assessed statistically using the standard Chi-square and also explored using subgroup analyses based on the different quantitative study designs included in this review.

#### Narrative Analysis

Where statistical pooling is not possible, the findings will be presented in a narrative form including tables and figures to aid in data presentation where appropriate. This will involve the aggregation or synthesis of conclusions to generate a set of statements that thematically represents such aggregation through accumulating and categorizing these conclusions on the basis of congruency in meaning. The following themes will be narratively reported in the report:

1. Influence of migration on allostatic load;
2. Longitudinal changes and temporal factors related to migration affecting the allostatic load;
3. Allostatic load parameters in migrant children;
4. Methods in measuring allostatic load parameters in migrant children.

## Results

At the time that this systematic review protocol was prepared, the researchers have conducted preliminary literature search suing the abovementioned search strategies. The results of such initial search are shown in Multimedia Appendix 2. This suggests that the search strategy is effective in supporting the objectives of this research. It is intended to replicate the search strategy in other databases. Appraisal, synthesis and analysis of the evidence will be finished by June 2018. It is projected that the manuscript that describes the results of this systematic review will be available by the last quarter of 2018.

## Discussion

Work on this systematic review was initiated last July 2017 and should come into completion by March 2018. Knowledge translation shall include collaboration with international and local affiliates including researchers, decision makers, service providers and migrant families. The major output document shall be the main systematic review which will synthesize the works of the authors, which is aimed to be presented in several media platforms: 1) dissemination of outputs through websites; 2) sharing of outputs through local and international news media outfits; 3) organization of a dialogue between researchers and representatives from embassies and consulates; 4) submission of the article in a peer-reviewed and open-access journal; and 5) presentations at local and international conferences.

We anticipate a limited number of studies on this subject matter, probably due to the overarching theoretical framework applied to describing the influences of migration and the use of allostatic load as parameters of change. Hence, it may likely that we include all available studies despite issues on quality. However, these will be accounted for and reported in succinct detail with respect to the risk of bias involved. Furthermore, the authors will exert the available effort and resources to find other sources of relevant data. Our review may thus be able to map out the scope of the available extant literature relevant to this matter.

The results of this review will not only provide insight on the previously unidentified neurophysiological mechanism underlying the adaptive processes related to migration to a foreign environment of resettlement among children, but has profound implications for understanding the unique health and well-being conditions and needs of this growing population. The potential findings of our review of the evidence can influence the development of innovative evidence-informed evaluation and intervention programs that are empirically relevant and sensitive to the needs of migrant children. Furthermore, the findings of this review have the prospective impetus in guiding the local government to design and enact health and sociopolitical policies responsive of migrant children's current contexts.

## Appendix

Multimedia Appendix 1 PRISMA-P checklist.

Multimedia Appendix 2 Sample search terms.

Multimedia Appendix 3 Sample flow of the study.

Multimedia Appendix 4 Appraisal instruments.

Multimedia Appendix 5 Levels of evidence.

Multimedia Appendix 6 Data extraction tools.

Multimedia Appendix 7 Risk of bias tool.

## Abbreviations

JBI-MAStARI: Joanna Briggs Institute Meta-Analysis of Statistics Assessment and Review Instrument
NHMRC: National Health and Medical Research Council
PRISMA-P: Preferred Reporting Items for Systematic Review and Meta-Analysis Protocols
