# Supplementary material for: Migration Influences on the Allostatic Load of Children: Systematic Review Protocol
Source: JMIR Res Protoc. 2018 Jan 30;7(1):e29. doi: 10.2196/resprot.8332 (PMC5811654; doi:10.2196/resprot.8332)
Supplement: Multimedia Appendix 2 [file resprot_v7i1e29_app2.pdf]

## Appendix 1.

Table 1. Sample MEDLINE search strategy and preliminary results

| Search # | Search Terms | Results |
|---|---|---|
| 1 | allostasis.mp. [mp=title, abstract, original title, name of substance word, subject heading word, keyword heading word, protocol supplementary concept word, rare disease supplementary concept word, unique identifier, synonyms] | 724 |
| 2 | allostatic.mp. [mp=title, abstract, original title, name of substance word, subject heading word, keyword heading word, protocol supplementary concept word, rare disease supplementary concept word, unique identifier, synonyms] | 1007 |
| 3 | allostatic load.mp. [mp=title, abstract, original title, name of substance word, subject heading word, keyword heading word, protocol supplementary concept word, rare disease supplementary concept word, unique identifier, synonyms] | 1007 |
| 4 | allostatic load.mp. [mp=title, abstract, original title, name of substance word, subject heading word, keyword heading word, protocol supplementary concept word, rare disease supplementary concept word, unique identifier, synonyms] | 1302 |
| 5 | migration.mp. [mp=title, abstract, original title, name of substance word, subject heading word, keyword heading word, protocol supplementary concept word, rare disease supplementary concept word, unique identifier, synonyms] | 218299 |
| 6 | migrant.mp. [mp=title, abstract, original title, name of substance word, subject heading word, keyword heading word, protocol supplementary concept word, rare disease supplementary concept word, unique identifier, synonyms] | 8132 |
| 7 | immigration.mp. [mp=title, abstract, original title, name of substance word, subject heading word, keyword heading word, protocol supplementary concept word, rare disease supplementary concept word, unique identifier, synonyms] | 30601 |
| 8 | immigrant.mp. [mp=title, abstract, original title, name of substance word, subject heading word, keyword heading word, protocol supplementary concept word, rare disease supplementary concept word, unique identifier, synonyms] | 11615 |
| 9 | migra*.mp. [mp=title, abstract, original title, name of substance word, subject heading word, keyword heading word, protocol supplementary concept word, rare disease supplementary concept word, unique identifier, synonyms] | 323518 |
| 10 | children.mp. [mp=title, abstract, original title, name of substance word, subject heading word, keyword heading word, protocol supplementary concept word, rare disease supplementary concept word, unique identifier, synonyms] | 911996 |
| 11 | child*.mp. [mp=title, abstract, original title, name of substance word, subject heading word, keyword heading word, protocol supplementary concept word, rare disease supplementary concept word, unique identifier, synonyms] | 2212191 |
| 12 | 1 or 2 or 3 or 4 | 1302 |
| 13 | 5 or 6 or 7 or 8 or 9 | 347902 |
| 14 | 10 or 11 | 2212191 |
| 15 | 12 and 13 | 33 |

| | | |
|---|---|---|
| 16 | 12 and 13 | 200 |
| 17 | 13 and 14 | 24849 |
| 18 | 12 and 13 and 14 | 4 |
