# Supplementary figures and images for: Migration Influences on the Allostatic Load of Children: Systematic Review Protocol
Source: JMIR Res Protoc. 2018 Jan 30;7(1):e29. doi: 10.2196/resprot.8332 (PMC5811654; doi:10.2196/resprot.8332)

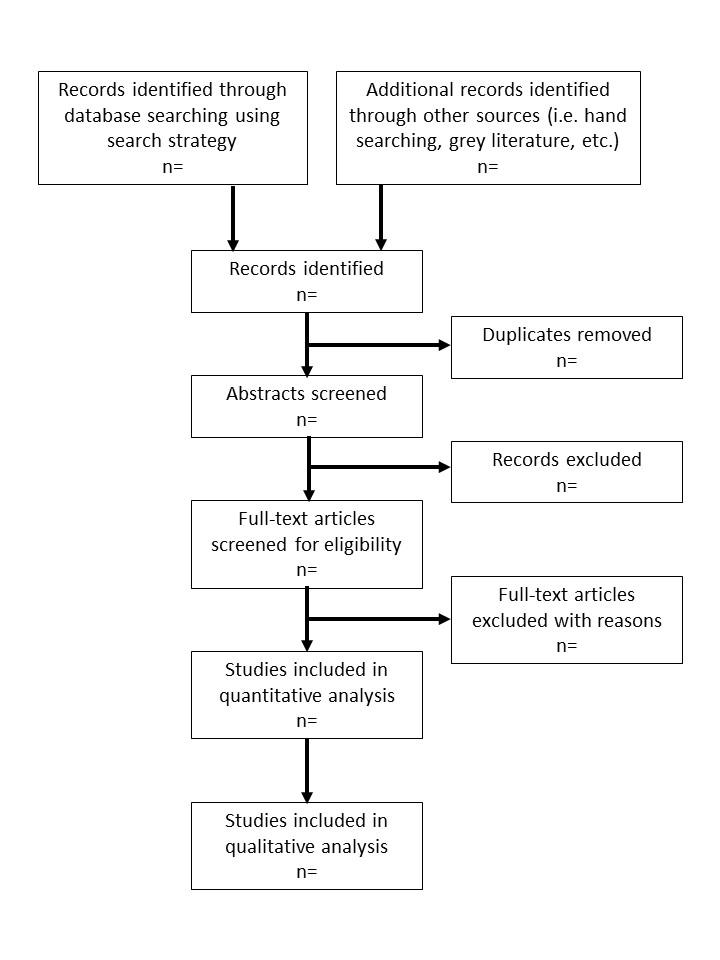

Supplement: Multimedia Appendix 3 [file resprot_v7i1e29_app3.jpg]
