# Supplementary material for: Migration Influences on the Allostatic Load of Children: Systematic Review Protocol
Source: JMIR Res Protoc. 2018 Jan 30;7(1):e29. doi: 10.2196/resprot.8332 (PMC5811654; doi:10.2196/resprot.8332)
Supplement: Multimedia Appendix 4 [file resprot_v7i1e29_app4.pdf]

Appendix II: Appraisal Instruments

QARI Appraisal instrument (source: JBI, 2008)

JBI QARI Critical Appraisal Checklist for Interpretive & Critical Research

Reviewer _______________________ Date _______________________

Author _________________________ Year _______ Record Number _______

	Yes	No	Unclear	Not Applicable
1. Is there congruity between the stated philosophical perspective and the research methodology?	☐	☐	☐	☐
2. Is there congruity between the research methodology and the research question or objectives?	☐	☐	☐	☐
3. Is there congruity between the research methodology and the methods used to collect data?	☐	☐	☐	☐
4. Is there congruity between the research methodology and the representation and analysis of data?	☐	☐	☐	☐
5. Is there congruity between the research methodology and the interpretation of results?	☐	☐	☐	☐
6. Is there a statement locating the researcher culturally or theoretically?	☐	☐	☐	☐
7. Is the influence of the researcher on the research, and vice- versa, addressed?	☐	☐	☐	☐
8. Are participants, and their voices, adequately represented?	☐	☐	☐	☐
9. Is the research ethical according to current criteria or, for recent studies, and is there evidence of ethical approval by an appropriate body?	☐	☐	☐	☐
10. Do the conclusions drawn in the research report flow from the analysis, or interpretation, of the data?	☐	☐	☐	☐

Overall appraisal: ☐ Include ☐ Exclude ☐ Seek further info. ☐

Comments (Including reason for exclusion)

___

___

___

MAStARI Appraisal instrument (source: JBI, 2008)

JBI Critical Appraisal Checklist for Randomised Control / Pseudo-randomised Trial

Reviewer ________________________ Date ________________________

Author ________________________ Year _______ Record Number _______

	Yes	No	Unclear	Not Applicable
1. Was the assignment to treatment groups truly random?	☐	☐	☐	☐
2. Were participants blinded to treatment allocation?	☐	☐	☐	☐
3. Was allocation to treatment groups concealed from the allocator?	☐	☐	☐	☐
4. Were the outcomes of people who withdrew described and included in the analysis?	☐	☐	☐	☐
5. Were those assessing outcomes blind to the treatment allocation?	☐	☐	☐	☐
6. Were the control and treatment groups comparable at entry?	☐	☐	☐	☐
7. Were groups treated identically other than for the named interventions	☐	☐	☐	☐
8. Were outcomes measured in the same way for all groups?	☐	☐	☐	☐
9. Were outcomes measured in a reliable way?	☐	☐	☐	☐
10. Was appropriate statistical analysis used?	☐	☐	☐	☐
	☐	☐	☐	☐

Overall appraisal: Include ☐ Exclude ☐ Seek further info. ☐

Comments (Including reason for exclusion)

____

____

____

JBI Critical Appraisal Checklist for Descriptive / Case Series

Reviewer ______________________ Date ______________________

Author ________________________ Year _______ Record Number _______

	Yes	No	Unclear	Not Applicable
1. Was study based on a random or pseudo-random sample?	☐	☐	☐	☐
2. Were the criteria for inclusion in the sample clearly defined?	☐	☐	☐	☐
3. Were confounding factors identified and strategies to deal with them stated?	☐	☐	☐	☐
4. Were outcomes assessed using objective criteria?	☐	☐	☐	☐
5. If comparisons are being made, was there sufficient descriptions of the groups?	☐	☐	☐	☐
6. Was follow up carried out over a sufficient time period?	☐	☐	☐	☐
7. Were the outcomes of people who withdrew described and included in the analysis?	☐	☐	☐	☐
8. Were outcomes measured in a reliable way?	☐	☐	☐	☐
9. Was appropriate statistical analysis used?	☐	☐	☐	☐

Overall appraisal: Include ☐ Exclude ☐ Seek further info ☐

Comments (Including reason for exclusion)

____

____

____

JBI Critical Appraisal Checklist for Comparable Cohort/ Case Control

Reviewer _________________________ Date __________________________

Author ___________________________ Year _______ Record Number _______

	Yes	No	Unclear	Not Applicable
1. Is sample representative of patients in the population as a whole?	☐	☐	☐	☐
2. Are the patients at a similar point in the course of their condition/illness?	☐	☐	☐	☐
3. Has bias been minimised in relation to selection of cases and of controls?	☐	☐	☐	☐
4. Are confounding factors identified and strategies to deal with them stated?	☐	☐	☐	☐
5. Are outcomes assessed using objective criteria?	☐	☐	☐	☐
6. Was follow up carried out over a sufficient time period?	☐	☐	☐	☐
7. Were the outcomes of people who withdrew described and included in the analysis?	☐	☐	☐	☐
8. Were outcomes measured in a reliable way?	☐	☐	☐	☐
9. Was appropriate statistical analysis used?	☐	☐	☐	☐

Overall appraisal: Include ☐ Exclude ☐ Seek further info. ☐

Comments (Including reason for exclusion)

____

____

____
