# Supplementary material for: Migration Influences on the Allostatic Load of Children: Systematic Review Protocol
Source: JMIR Res Protoc. 2018 Jan 30;7(1):e29. doi: 10.2196/resprot.8332 (PMC5811654; doi:10.2196/resprot.8332)
Supplement: Multimedia Appendix 5 [file resprot_v7i1e29_app5.pdf]

Appendix IV. NHMRC Levels of Evidence (NHMRC, 2009)

Table 1 NHMRC Evidence Hierarchy: designations of 'levels of evidence' according to type of research question (including explanatory notes)

Level	Intervention [1]	Diagnostic accuracy [2]	Prognosis	Aetiology [3]	Screening Intervention
I [4]	A systematic review of level II studies	A systematic review of level II studies	A systematic review of level II studies	A systematic review of level II studies	A systematic review of level II studies
II	A randomised controlled trial	A study of test accuracy with: an independent, blinded comparison with a valid reference standard,[5] among consecutive persons with a defined clinical presentation[6]	A prospective cohort study[7]	A prospective cohort study	A randomised controlled trial
III-1	A pseudorandomised controlled trial (i.e. alternate allocation or some other method)	A study of test accuracy with: an independent, blinded comparison with a valid reference standard,[5] among non-consecutive persons with a defined clinical presentation[6]	All or none[8]	All or none[8]	A pseudorandomised controlled trial (i.e. alternate allocation or some other method)
III-2	A comparative study with concurrent controls:<br>• Non-randomised, experimental trial[9]<br>• Cohort study<br>• Case-control study<br>• Interrupted time series with a control group	A comparison with reference standard that does not meet the criteria required for Level II and III-1 evidence	Analysis of prognostic factors amongst persons in a single arm of a randomised controlled trial	A retrospective cohort study	A comparative study with concurrent controls:<br>• Non-randomised, experimental trial<br>• Cohort study<br>• Case-control study
III-3	A comparative study without concurrent controls:<br>• Historical control study<br>• Two or more single arm study[10]<br>• Interrupted time series without a parallel control group	Diagnostic case-control study[6]	A retrospective cohort study	A case-control study	A comparative study without concurrent controls:<br>• Historical control study<br>• Two or more single arm study
IV	Case series with either post-test or pre-test/post-test outcomes	Study of diagnostic yield (no reference standard)[11]	Case series, or cohort study of persons at different stages of disease	A cross-sectional study or case series	Case series
