# Supplementary material for: Migration Influences on the Allostatic Load of Children: Systematic Review Protocol
Source: JMIR Res Protoc. 2018 Jan 30;7(1):e29. doi: 10.2196/resprot.8332 (PMC5811654; doi:10.2196/resprot.8332)
Supplement: Multimedia Appendix 6 [file resprot_v7i1e29_app6.pdf]

Appendix III: Data extraction instruments

QARI data extraction instrument (source: JBI, 2008)

JBI QARI Data Extraction Form for Interpretive & Critical Research

Reviewer ______________________ Date ______________________

Author ______________________ Year ______________________

Journal ______________________ Record Number ______________________

Study Description

Methodology

____

Method

____

Phenomena of interest

____

Setting

____

Geographical

____

Cultural

____

Participants

____

Data analysis

____

Authors Conclusions

____

Comments

____

Complete Yes ☐ No ☐

Findings	Illustration from Publication (page number)	Evidence		
		Unequivocal	Credible	Unsupported

Extraction of findings complete Yes ☐ No ☐

MAStARI data extraction instrument (source: JBI, 2008)

JBI Data Extraction Form for Experimental / Observational Studies

Reviewer ______________________ Date ______________________

Author ______________________ Year ______________________

Journal ______________________ Record Number ______________________

Study Method

RCT	☐	Quasi-RCT	☐	Longitudinal	☐
Retrospective	☐	Observational	☐	Other	☐

Participants

Setting ______________________

Population ______________________

Sample size

Group A ______________________ Group B ______________________

Interventions

Intervention A ______________________

____

Intervention B ______________________

____

Authors Conclusions:

____

____

____

Reviewers Conclusions:

____

____

____

Study results

Dichotomous data

Outcome	Intervention ( ) number / total number	Intervention ( ) number / total number

Continuous data

Outcome	Intervention ( ) number / total number	Intervention ( ) number / total number
