# Supplementary material for: Migration Influences on the Allostatic Load of Children: Systematic Review Protocol
Source: JMIR Res Protoc. 2018 Jan 30;7(1):e29. doi: 10.2196/resprot.8332 (PMC5811654; doi:10.2196/resprot.8332)
Supplement: Multimedia Appendix 7 [file resprot_v7i1e29_app7.pdf]

Appendix V. Cochrane Risk of Bias Tool (Cochrane, 2011)

AUB KQ1 Risk of Bias Assessment (Reference ID #)

Domain	Description	High Risk of Bias	Low Risk of Bias	Unclear Risk of Bias	Reviewer Assessment	Reviewer Comments
Selection bias ***Random sequence generation***	Described the method used to generate the allocation sequence in sufficient detail to allow an assessment of whether it should produce comparable groups	Selection bias (biased allocation to interventions) due to inadequate generation of a randomized sequence	Random sequence generation method should produce comparable groups	Not described in sufficient detail	**High** **Low** **Unclear**	
Selection bias ***Allocation concealment***	Described the method used to conceal the allocation sequence in sufficient detail to determine whether intervention allocations could have been foreseen before or during enrollment	Selection bias (biased allocation to interventions) due to inadequate concealment of allocations prior to assignment	Intervention allocations likely could not have been foreseen in before or during enrollment	Not described in sufficient detail	**High** **Low** **Unclear**	
Reporting bias ***Selective reporting***	Stated how the possibility of selective outcome reporting was examined by the authors and what was found	Reporting bias due to selective outcome reporting	Selective outcome reporting bias not detected	Insufficient information to permit judgment†	**High** **Low** **Unclear**	
Other bias ***Other sources of bias***	Any important concerns about bias not addressed above*	Bias due to problems not covered elsewhere in the table	No other bias detected	There may be a risk of bias, but there is either insufficient information to assess whether an important risk of bias exists or insufficient rationale or evidence that an identified problem will introduce bias	**High** **Low** **Unclear**	

* If particular questions/entries were pre-specified in the study's protocol, responses should be provided for each question/entry.
† It is likely that the majority of studies will fall into this category.

Assess each main or class of outcomes for each of the following. Indicate the specific outcome.

AUB KQ1 Risk of Bias Assessment (Reference ID #)

Outcome:

Domain	Description	High Risk of Bias	Low Risk of Bias	Unclear Risk of Bias	Reviewer Assessment	Reviewer Comments
Performance bias ***Blinding (participants and personnel)***	Described all measures used, if any, to blind study participants and personnel from knowledge of which intervention a participant received. Provided any information relating to whether the intended blinding was effective.	Performance bias due to knowledge of the allocated interventions by participants and personnel during the study.	Blinding was likely effective.	Not described in sufficient detail	**High**<br>**Low**<br>**Unclear**	
Detection bias ***Blinding (outcome assessment)***	Described all measures used, if any, to blind outcome assessors from knowledge of which intervention a participant received. Provided any information relating to whether the intended blinding was effective.	Detection bias due to knowledge of the allocated interventions by outcome assessors.	Blinding was likely effective.	Not described in sufficient detail	**High**<br>**Low**<br>**Unclear**	
Attrition bias ***Incomplete outcome data***	Described the completeness of outcome data for each main outcome, including attrition and exclusions from the analysis. Stated whether attrition and exclusions were reported, the numbers in each intervention group (compared with total randomized participants), reasons for attrition/exclusions where reported.	Attrition bias due to amount, nature or handling of incomplete outcome data.	Handling of incomplete outcome data was complete and unlikely to have produced bias	Insufficient reporting of attrition/exclusions to permit judgment (e.g., number randomized not stated, no reasons for missing data provided)	**High**<br>**Low**<br>**Unclear**	
